# A T-shape diphosphinoborane palladium(0) complex

**DOI:** 10.3762/bjoc.12.152

**Published:** 2016-07-22

**Authors:** Patrick Steinhoff, Michael E Tauchert

**Affiliations:** 1 Institute of Inorganic Chemistry, RWTH Aachen University, Landoltweg 1, D-52074 Aachen, Germany

**Keywords:** ambiphilic ligand, coordination chemistry, diphosphinoborane, organometallics, palladium

## Abstract

The reaction of CpPd(η*^3^*-C_3_H_5_) with the new diphosphinoborane ligand derivative (*o*-PCy_2_-C_6_H_4_)_2_BPh **^Cy^DPB^Ph^** affords the T-shape complex (**^Cy^DPB^Ph^**)Pd(0) **9**, which was characterized by X-ray analysis.

## Introduction

**Figure 1:**
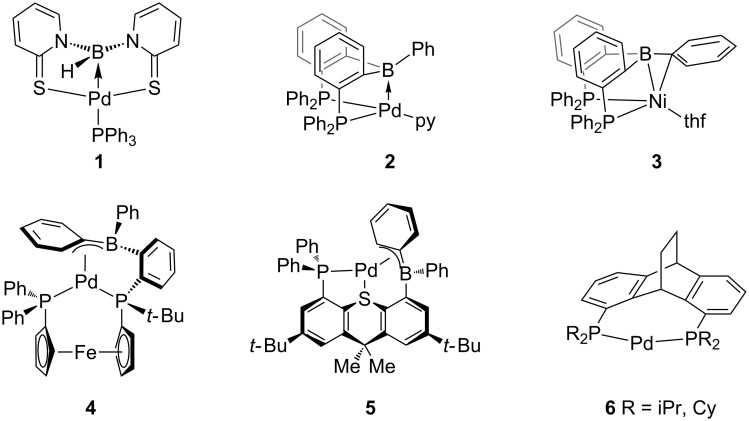
Selected M→B coordination modes **1**–**5** [6–10] and Hofmann’s Rucaphos complex **6** [11].

The amplification of traditional bidentate chelating L_2_-type ligands with a tethered borane functionality (e.g., Bourissou’s diphospinoborane (*o*-PR_2_-C_6_H_4_)_2_BR’ ligand **^R^DPB^R’^**) has received considerable attention [1–3], with first catalytic applications emerging [4]. The acyclic boron group in these ligands can adopt a variety of coordination modes (Figure 1) [5].

The borane can act as a σ-acceptor ligand in case of η^1^-B coordination (e.g., **1** [6] and **2** [7]), or as a boron containing π-ligand adopting η^2^-B,C (**3**) [8] or η^3^-B,C,C coordination (**4** and **5**) [5,9–10]. Changes of the hapticity appear to have significant influence onto the reactivity of the coordinated transition metal towards substrates [8]. For zerovalent palladium complexes only few examples featuring a η^1^-type Pd→B interaction have been reported [6–7]. However, these complexes require phosphines or pyridines as a stabilizing co-ligand, which can act as an inhibitor in catalytic transformations [7]. Similarly, monometallic 14 VE palladium complexes featuring a chelating diphosphine, such as in Hofmanns Rucaphos complexes **6**, are very scarce [11]. While the dative Pd→B bond is strong in zerovalent Pd(0) **DPB** complexes such as **2**, only weak Pd→B interactions have been observed for the respective Pd(II) complexes [7,12]. Discrimination by the borane functionality between the oxidations states Pd(0)/Pd(II) is of potential interest for organometallic transformations involved in homogeneous catalysis, such as the reductive elimination. Here we report the synthesis of the diphosphinoborane (*o*-PCy_2_-C_6_H_4_)_2_BPh ligand **^Cy^DPB^Ph^**. **^Cy^DPB^Ph^** reacts with CpPd(η^3^-C_3_H_5_) yielding monometallic zerovalent palladium complex **9** featuring a distinct η^1^*-*B coordination mode, without the need of a stabilizing co-ligand.

## Findings

**Scheme 1:**
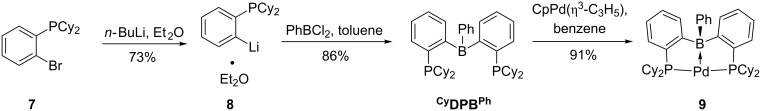
Synthesis of diphosphinoborane **^Cy^DPB^Ph^** and complex **9**.

For the synthesis of **^Cy^DPB^Ph^** we adapted the known reaction sequence for the production of Bourissou’s (*o*-PPh_2_-C_6_H_4_)_2_BPh ligand **^Ph^DPB^Ph^** (Scheme 1) [13–14].

Starting material (2-bromophenyl)dicyclohexylphosphine (**7**) was produced by palladium catalyzed coupling of dicyclohexylphosphine with 1-iodo-2-bromobenzene [15]. Phosphine **7** was lithiated in diethyl ether with *n-*BuLi [16–17], affording the diethyl ether adduct **8**. Reaction of **8** with 0.5 equiv of PhBCl_2_ in toluene at −78 °C produced the desired ligand **^Cy^DBP^Ph^** in 86% isolated yield. Typical resonances for a DPB ligand were observed in the ^31^P NMR spectrum at δ 1.70 and in the ^11^B NMR spectrum at δ 41 (w_1/2_ = 1300 ± 120 Hz), which are indicative for a dynamic P→B bond in solution [18].

**Figure 2:**
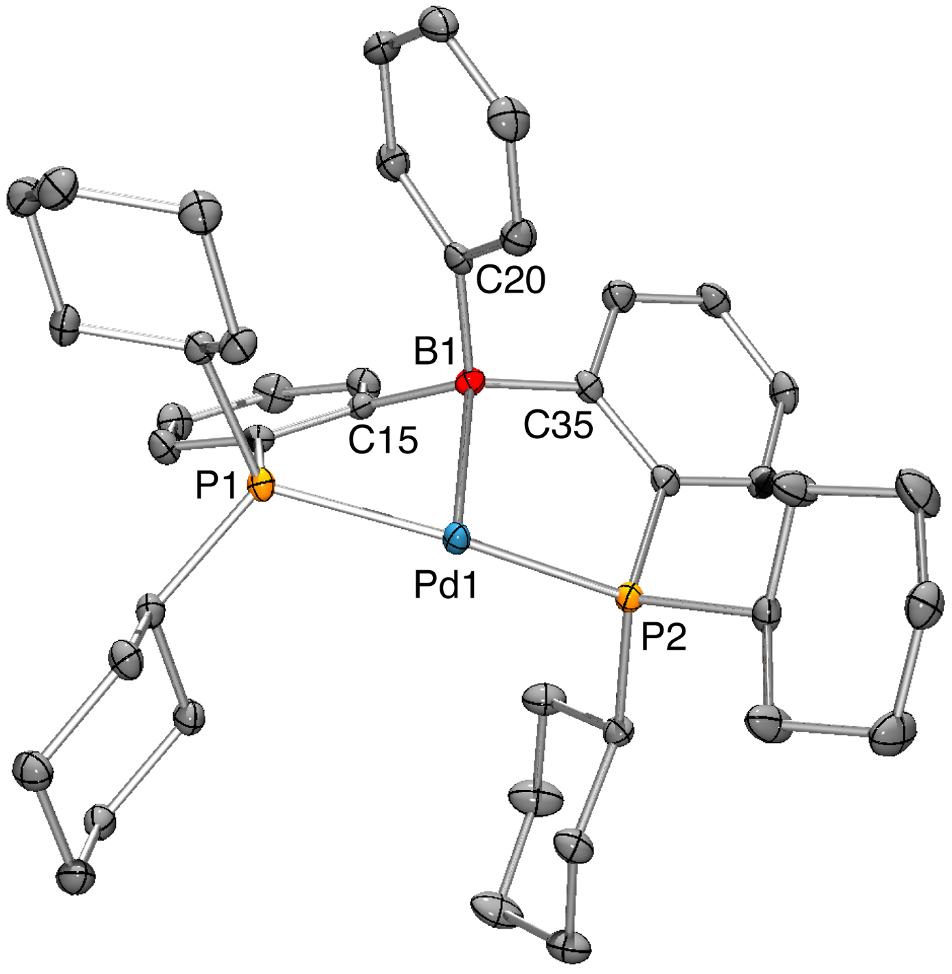
Thermal ellipsoid plots of complex **9** at the 50% probability level. H atoms and one molecule of hexane have been omitted for clarity. Selected interatomic distances (Å) and angles (°): Pd1–B1 2.243(2), Pd1–P1 2.2761(6), Pd1–P2 2.3084(6), B1–Pd1–P1 85.82(6), B1–Pd1–P2 82.49(6), P1–Pd1–P2 157.72(2), C15–B1–C20 110.94(18), C15–B1–C35 116.58(18), C20–B1–C35 112.56(18).

**^Cy^DPB^Ph^** was reacted with 1 equiv of CpPd(η^3^-C_3_H_5_) in benzene. Complete conversion towards complex **9** with equimolar formation of 5-allylcyclopenta-1,3-diene was reached within 18 h at 50 °C. Complex **9** showed a singlet resonance at δ 41.0 in the ^31^P NMR spectrum and a broad resonance at δ 22 (w_1/2_ = 800 ± 50 Hz) in the ^11^B NMR spectrum. High field shift and narrowing of the ^11^B NMR with respect to the free **^Cy^DPB^Ph^** ligand indicated the presence of a strong dative Pd(0)→B bond [7]. Despite the absence of a stabilizing co-ligand, we found complex **9** to be very stable in solution. The coordinating properties of **^Cy^DPB^Ph^** deviate from those observed for its aryl derivatives (**^Ph^DPB^Ph^** ((*o*-PPh_2_-C_6_H_4_)_2_BPh) and **^Ph^DPB^Mes^** ((*o*-PPh_2_-C_6_H_4_)_2_B(Mes))). For these ligands the reaction with one equivalent of CpPd(η^3^-C_3_H_5_) leads to 50% consumption of CpPd(η^3^-C_3_H_5_) with simultaneous formation of 5-allylcyclopenta-1,3-diene, but complete conversion of the ligand pointing towards the formation of a bisligand complex (DPB)_2_Pd [7]. Unlike complex **2** we were unable to form a pyridine adduct complex by treatment of **9** with 10 equiv of pyridine. Single crystals of complex **9** suitable for X-ray diffraction analysis were grown from hexane (Figure 2).

The solid-state structure of **9** displayed a slightly distorted T-shape geometry around the palladium center. A short Pd1–B1 distance of 2.243(2) Å (cf. complex **2**: 2.194(3) Å) and a significant pyramidalization at the boron center (ΣB_α_ = 341°) is observed, indicating a strong Pd(0)→B bond. The distance between C20 and Pd1 was found to be 3.0805(22) Å. The η^1^-B coordination mode was well reproduced by DFT calculations (Supporting Information File 1). DFT calculations predict T-shape complexes with an almost linear P–Pd–P angle for model complexes (PMe_3_)_2_Pd → EX_3_ (E = B; X = H, F, Cl, Br, I) [17]. In complex **9** the *trans*-coordinated palladium center featured an obtuse P1–Pd1–P2 angle of 157.72(2)°.

## Conclusion

In conclusion we synthesized the zerovalent palladium complex [{(*o*-PCy_2_-C_6_H_4_)_2_BPh}Pd(0)] **9**. Complex **9** supplements the few known examples (e.g., **6** [11]) of 14 VE palladium complexes bearing a chelating diphosphine ligand by introduction of a borane acceptor functionality.

## Supporting Information

File 1 Experimental procedures and characterization data; crystallographic information for **9**; ^1^H, ^11^B, ^13^C and ^31^P NMR spectra.

File 2 CIF file of **9**, CCDC 1471929.

## References

[1] Fontaine F-G, Boudreau J, Thibault M-H (2008). Eur J Inorg Chem.

[2] Amgoune A, Bourissou D (2011). Chem Commun.

[3] Kameo H, Nakazawa H (2013). Chem – Asian J.

[4] Bouhadir G, Bourissou D (2016). Chem Soc Rev.

[5] Emslie D J H, Cowie B E, Kolpin K B (2012). Dalton Trans.

[6] Zech A, Haddow M F, Othman H, Owen G R (2012). Organometallics.

[7] Schindler T, Lux M, Peters M, Scharf L T, Osseili H, Maron L, Tauchert M E (2015). Organometallics.

[8] Harman W H, Peters J C (2012). J Am Chem Soc.

[9] Emslie D J H, Harrington L E, Jenkins H A, Robertson C M, Britten J F (2008). Organometallics.

[10] Cowie B E, Emslie D J H (2015). Organometallics.

[11] Schnetz T, Röder M, Rominger F, Hofmann P (2008). Dalton Trans.

[12] Bontemps S, Sircoglou M, Bouhadir G, Puschmann H, Howard J A K, Dyer P W, Miqueu K, Bourissou D (2008). Chem – Eur J.

[13] Sircoglou M, Bontemps S, Mercy M, Saffon N, Takahashi M, Bouhadir G, Maron L, Bourissou D (2007). Angew Chem, Int Ed.

[14] Conifer C M, Law D J, Sunley G J, White A J P, Britovsek G J P (2011). Organometallics.

[15] Murata M, Buchwald S L (2004). Tetrahedron.

[16] Harder S, Brandsma L, Kanters J A, Duisenberg A, van Lenthe J H (1991). J Organomet Chem.

[17] Goedecke C, Hillebrecht P, Uhlemann T, Haunschild R, Frenking G (2009). Can J Chem.

[18] Bontemps S, Bouhadir G, Dyer P W, Miqueu K, Bourissou D (2007). Inorg Chem.

