# Supplementary material for: A T-shape diphosphinoborane palladium(0) complex
Source: Beilstein J Org Chem. 2016 Jul 22;12:1573–6. doi: 10.3762/bjoc.12.152 (PMC4979764; doi:10.3762/bjoc.12.152)
Supplement: File 1 — Experimental procedures and characterization data; crystallographic information for 9; 1H, 11B, 13C and 31P NMR spectra. [file Beilstein_J_Org_Chem-12-1573-s001.pdf]

Supporting Information
for
A T-shape diphosphinoborane palladium(0) complex

Patrick Steinhoff[1] and Michael E. Tauchert*[1]

Address: [1]Institute of Inorganic Chemistry, RWTH Aachen University, Landoltweg 1, D-52074 Aachen, Germany

Email: Michael E. Tauchert - Michael.Tauchert@ac.rwth-aachen.de

* Corresponding author

Experimental procedures and characterization data; crystallographic information for 9; ^{1}H, ^{11}B, ^{13}C and ^{31}P NMR spectra

General information S1
Single crystal X-ray diffraction S2
DFT calculation S3
Syntheses S4
NMR spectra S6
References S11

General information

All manipulations were carried out under an inert argon atmosphere using either standard Schlenk technique or a MBraun Glovebox. Et_2O, pentane, toluene, DCM and THF were taken from a solvent purification system (M. Braun, SPS 800). Benzene, 1,4-dioxane and *n*-hexane were dried over sodium and distilled under an argon atmosphere. Deuterated solvents were degassed using the freeze pump thaw technique and stored over molecular sieves (4 Å). Cyclopentadienyl allyl palladium [1] and (2-bromophenyl)dicyclohexylphosphine **7** [2] were synthesized following published procedures. NMR spectra were recorded with a Bruker Avance II 400.13 MHz spectrometer at room temperature in a Wilmad® quick pressure valve NMR tube. The ^{1}H and ^{13}C NMR spectra were referenced to residual solvent resonances [3] ^{11}B, ^{13}C, ^{19}F and ^{31}P NMR-spectra were recorded broadband decoupled. If purchased, all chemicals were used as delivered with no further purification.

Single crystal X-ray diffraction

Mo Kα radiation (λ = 0.71073 Å) was produced from an Incoatec I-μS microsource equipped with multilayer optics. Data were collected at 100(2) K on a Bruker D8 goniometer equipped with an Apex CCD detector. Temperature stability during data collection was realized by an Oxford Cryosystems 700 controller. Integration was performed by the SAINT software [4]. The SADABS software [5] was used for multi-scan absorption correction. Using Olex2 [6], the structure was solved with the ShelXS [7] structure solution program employing direct methods. Refinement was performed with the XL [8] refinement package using least squares minimization. These data can be obtained free of charge from The Cambridge Crystallographic Data Centre via www.ccdc.cam.ac.uk/data_request/cif

Identification code	CCDC 1471929 (complex **9**)
Empirical formula	$C_{48}H_{71}BP_2Pd$
Formula weight	827.20
Temperature/K	100
Crystal system	monoclinic
Space group	$P2_1/c$
a/Å	11.3000(10)
b/Å	14.4355(13)
c/Å	26.239(2)
α/°	90.00
β/°	94.704(2)
γ/°	90.00
Volume/Å^3	4265.7(7)
Z	4
ρ_{calc}g/cm^3	1.288
μ/mm^{-1}	0.542
F(000)	1760.0
Crystal size/mm^3	0.61 × 0.36 × 0.28
Radiation	MoKα (λ = 0.71073)
2Θ range for data collection/°	3.22 to 53.1
Index ranges	-14 ≤ h ≤ 14, -18 ≤ k ≤ 18, -32 ≤ l ≤ 32
Reflections collected	51374
Independent reflections	8861 [R_{int} = 0.0625, R_{sigma} = 0.0421]
Data/restraints/parameters	8861/0/471
Goodness-of-fit on F^2	1.042
Final R indexes [I>=2σ (I)]	R_1 = 0.0323, wR_2 = 0.0742
Final R indexes [all data]	R_1 = 0.0408, wR_2 = 0.0784
Largest diff. peak/hole / e Å^{-3}	0.62/-0.38

DFT calculation

Geometry optimization of complex **9** was carried out without any symmetry restrictions. The calculation was performed using Turbomole 6.5 [9]. RI-DFT calculation was carried out using BP86/def-SV(P).

Bond	distance or angle (XRD)	distance or angle (DFT)
Pd1–P1	2.2766(6) Å	2.277 Å
Pd1–P2	2.3082(6) Å	2.308 Å
Pd1–B1	2.244(2) Å	2.243 Å
C20–B1	1.614(3) Å	1.615 Å
C35–B1	1.621(3) Å	1.621 Å
C15–B1	1.610(3) Å	1.611 Å
P1–Pd1–P2	157.73(2)°	157.7°
C15–B1–C35	116.64(18)°	116.7°
C15–B1–C20	110.86(18)°	110.8°
C20–B1–C35	112.70(17)°	112.7°

Syntheses

(2-(Dicyclohexylphosphino)phenyl)lithium·Et$_2$O (8). *n*-BuLi (7.5 mL; 1.6 M; 12 mmol; 1.3 equiv.) was added to a solution of (2-bromophenyl)dicyclohexylphosphine **7** (3.253 g; 9.239 mmol; 1 equiv) in ether (15 mL) at −78 °C. After stirring the solution at −78 °C (1.5 h) and then at room temperature (0.5 h), a white precipitate was formed. The product was isolated by filtration and washing of the residue with Et$_2$O. Yield: 2.395 g (6.760 mmol; 73%).

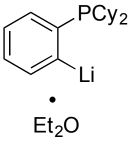

8

^{1}H NMR (THF-*d*$_8$, 400 MHz): δ = 7.72 (m, 1H, Ar-*H*); 7.03 (m, 1H, Ar-*H*); 6.63-6.73 (m, 2H, Ar-*H*); 3.39 (q, 4H, (CH$_3$C*H*$_2$)$_2$O); 1.93 – 1.70 (m, 6H, Cy-*H* and residual THF-*d*$_8$ signal); 1.71 – 1.58 (m, 4H, Cy-*H*); 1.35 – 0.95 (m, 18H, Cy-*H* and (C*H*$_3$CH$_2$)$_2$O) ppm. **^{31}P NMR** (THF-*d*$_8$, 162 MHz): δ = 3.96 (s) ppm. **^{7}Li NMR** (THF-*d*$_8$, 156 MHz): 1.41 (s) ppm.

((Phenylboranediyl)bis(2,1-phenylene))bis(dicyclohexylphosphine) (^{Cy}DPB^{Ph}). To a solution of (2-(dicyclohexylphosphino)phenyl)lithium·Et$_2$O (**8**) (1.999 g; 5.640 mmol; 1 equiv) in toluene (20 mL) cooled to −78 °C, a standard solution of PhBCl$_2$ in toluene (5.64 mL; 2.82 mmol; 0.50 M; 0.5 equiv) was added drop wise over a period of 4 h. After stirring for 1 h at −78 °C, all volatiles were removed in vacuo. The solid was solubilized in DCM, the salts were filtered off, and volatiles were removed under reduced pressure. **^{Cy}DPB^{Ph}** was obtained as a colorless powder by washing the resulting solid with pentane (3 × 5 mL) and removing all volatiles in vacuo. Yield: 1.547 g (2.44 mmol; 86%).

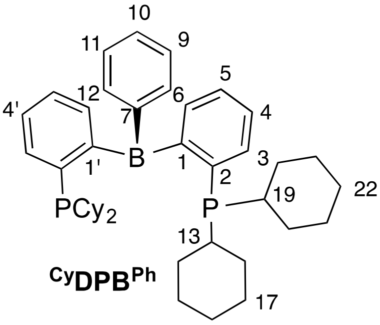

^{Cy}DPB^{Ph}

^{1}H NMR (DCM-*d*$_2$, 400 MHz): δ = 8.07 (d, 2H, J = 7.5 Hz, Ar-*H*); 7.43-7.35 (m, 4H, Ar-*H*); 7.28-7.20 (m, 4H, Ar-*H*); 7.16-7.11 (m, 3H, Ar-*H*); 1.88-1.78 (m, 4H, Cy-*H*); 1.74-1.59 (m, 8H, Cy-*H*); 1.59-1.46 (m, 8H, Cy-*H*); 1.46-1.37 (m, 4H, Cy-*H*); 1.18-0.96 (m, 16H, Cy-*H*); 0.93-0.81 (m, 4H, Cy-*H*) ppm. **^{11}B NMR** (DCM-*d*$_2$, 128 MHz): δ = 41 (s, $w_{1/2}$ = 1300 ± 120 Hz) ppm. **^{13}C NMR** (DCM-*d*$_2$, 101 MHz): δ = 160.87 (bs, 1C, C1 or C1’); 160.43 (bs, 1C, C1’ or C1); 148.55 (bs, 1C, C7); 136.76-136.57 (m, 3C); 135.257 (pt, 2C, J = 23.7 Hz, C2, C2;); 131.43 (s, 2C); 128.68 (s, 2C); 128.40 (s, 1C, C10); 127.37 (s, 2C); 126.41 (s, 2C); 35.76 (m$_c$, 4C, C13, C13’, C19, C19’); 30.47-30.07 (m, 8C, C14, C14’, C18, C18’, C20, C20’, C24, C24’); 27.88 (m$_c$, 4C); 27.71 (m$_c$, 4C); 26.88 (s, 4C, C16, C16’, C22, C22’) ppm. **^{31}P NMR** (DCM-*d*$_2$, 162 MHz): δ = 1.70 (s) ppm. **MS**: EI$^+$ m/z = 634.5 (M), 551.4 (M-Cy), 437.3 (M-PCy$_2$), 361.3 (M-PPhCy$_2$), 274.3 (PPhCy$_2$) and 192.2 (PCyPh). HRMS (EI): calculated for $C_{42}H_{57}BP_2$ (M) 634.40286; found 634.40260. **IR** (KBr): $\tilde{\nu}$ = 3388 (vs), 2922

(m), 2848 (w), 1628 (m), 1446 (vw), 1236 (vw), 521 (m) cm^{-1}. **M.P**. = 280 °C (decomposition).

Synthesis of {[(o-PCy$_2$C$_6$H$_4$)$_2$BPh]Pd(0)} (9). ^{Cy}**DPB**^{Ph} (500.2 mg, 788.1 µmol, 1 equiv) and CpPd-(η^3-C_3H_5) (167.7 mg, 787.7 µmol, 1 equiv) were solubilized in benzene (2 mL). After stirring for 18 h at 50 °C the volatiles were removed in vacuo. The product was obtained after washing the yellow residue three times with pentane (3 mL) and removing all volatiles in vacuo. Yield: 532.2 mg (718.1 µmol; 91%). Compound **9** may be crystalized from hexane or pentane. Crystals suitable for single crystal X-ray diffraction analysis were obtained by crystallization from hexane at 4 °C.

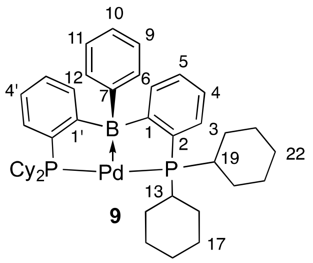

^{1}**H NMR** (benzene-d_6, 400 MHz): δ = 7.79 (d, 2H, J = 7.0 Hz, H3/H3'); 7.63 (dd, 2H, J = 8.1 Hz, J = 1.5 Hz, H6/H6'); 7.27 (pdt, 2H, J = 7.4 Hz, 3.0 Hz, Ar-*H*); 7.24-7.17 (m, 4H, Ar-*H*); 7.14-7.09 (m, 3H, Ar-*H*); 2.14 (ptt, 2H, J = 12.0 Hz, 2.6 Hz, H13 or H19); 2.09-1.97 (m, 6H, Cy-*H*); 1.83-1.75 (m, 2H, Cy-*H*); 1.72-1.47 (m, 14H, Cy-*H*); 1.46-0.91 (m, 20H, Cy-*H*) ppm. ^{11}**B NMR** (benzene-d_6, 128 MHz): δ = 22 (s, $w_{1/2}$ = 800 ± 50 Hz) ppm. ^{13}**C NMR** (benzene-d_6, 101 MHz): δ = 166.33 (bs, 2C, C1/C1'); 156.00 (bs, 1C, C7); 135.12 (s, 2C, C6/C6'); 134.82 (pt, 2C, J = 16.4 Hz, C2/C2'); 132.28 (pt, 2C, J = 10.8 Hz, C3/C3'); 129.99 (s, 2C); 128.71 (s, 2C); 126.82 (s, 2C); 125.74 (s, 1C, C10); 125.15 (t, 2C, J = 2.6 Hz, C4/C4'); 35.9 (pt, 2C, J = 9.3 Hz, C13'/C13); 35.66 (pt, 2C, J = 9.3 Hz, C13/C13'); 31.75 (pt, 2C, 4.5 Hz, C13/C13' or C19/C19'); 31.33 (pt, 2C, J = 4.0 Hz, C19/C19' or C13/C13'); 29.70 (s, 2C); 29.15 (s, 2C); 27.50 (pt, 2C, J = 6.6 Hz); 27.43 (pt, 2C, J = 5.1 Hz); 27.23 (pt, 2C, J = 5.8 Hz); 27.09 (pt, 2C, J = 6.8 Hz); 26.59 (s, 2C, C16/C16'); 26.29 (s, 2C, C16'/C16) ppm. ^{31}**P NMR** (benzene-d_6, 162 MHz): δ = 41.0 (s) ppm. **MS**: EI^+ m/z = 740.3 (M), 663.3 (M-Ph), 580.2 (M-Ph-Cy), 361.3 (M-PdPPhCy$_2$), 274.3 (PPhCy$_2$), 192.2 (PCyPh). **Combustion analysis** (batch crystalized from pentane): calculated for $C_{42}H_{57}BP_2Pd \bullet 0.5C_5H_{12}$ C 68.77%, H 8.17%; found: C 68.91%, H 8.092. **M.P**. = 126 °C (decomposition).

NMR Spectra

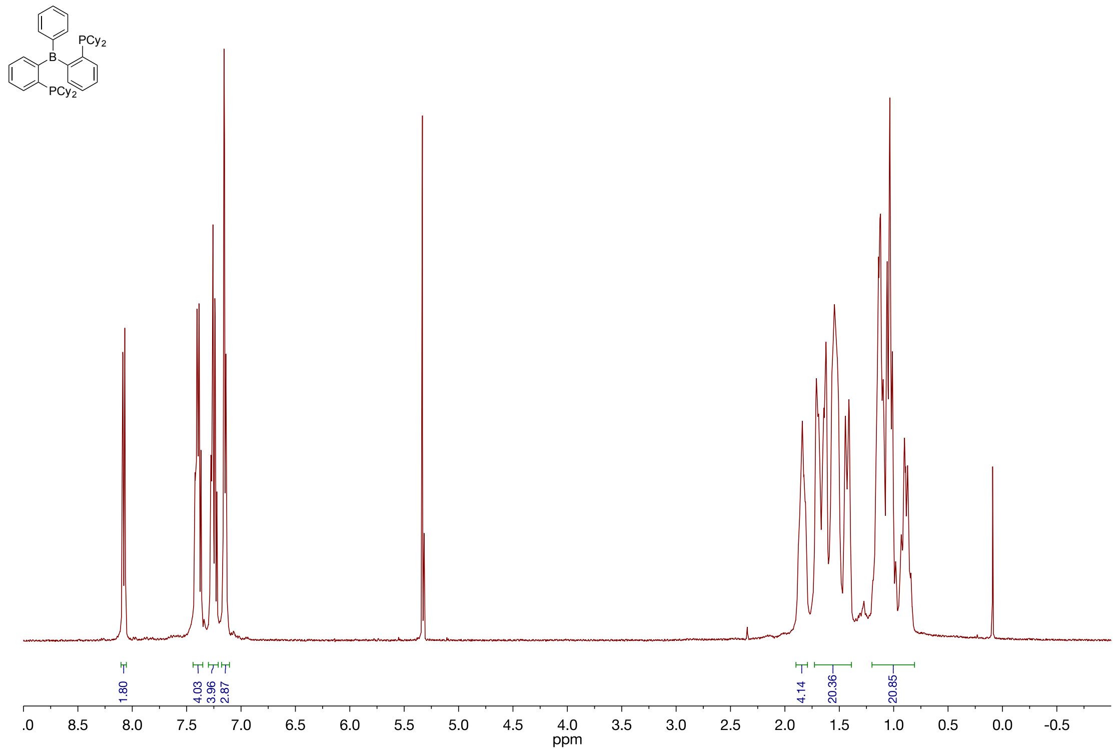

Fig. S1. ^{1}H NMR (400 MHz, dichloromethane-d^{2}) of ^{Cy}**DPB^{Ph}.**

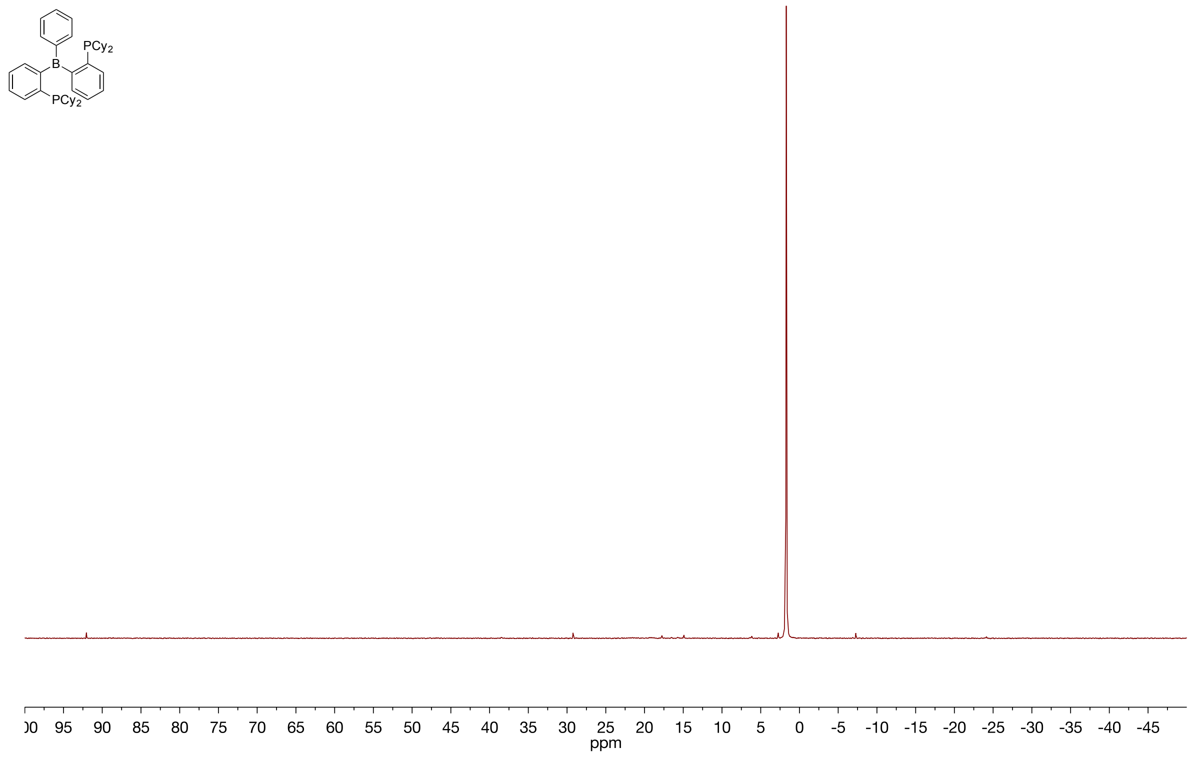

Fig. S2. ^{31}P NMR (162 MHz, dichloromethane-d^{2}) of ^{Cy}**DPB^{Ph}.**

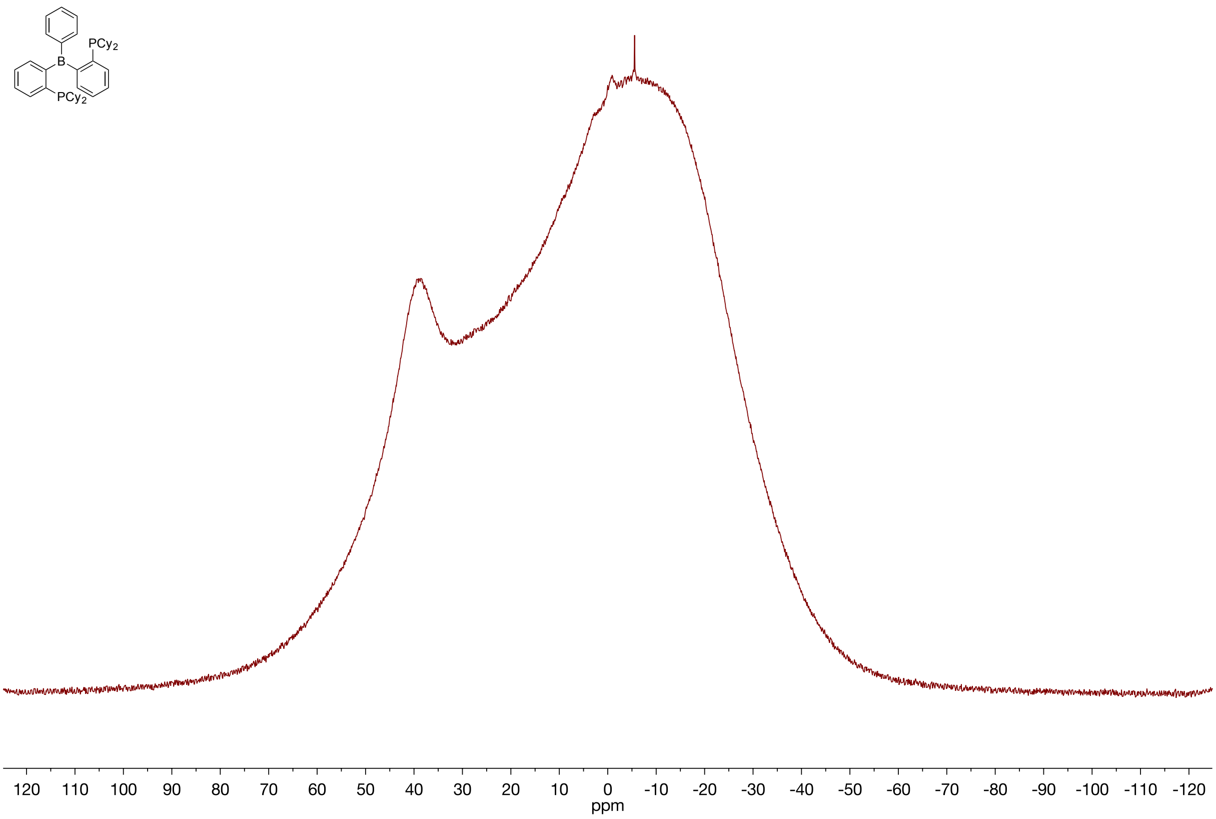

Fig. S3. ^{11}B NMR (128 MHz, dichloromethane-d^{2}) of ^{Cy}**DPB**$^{\mathbf{Ph}}$.

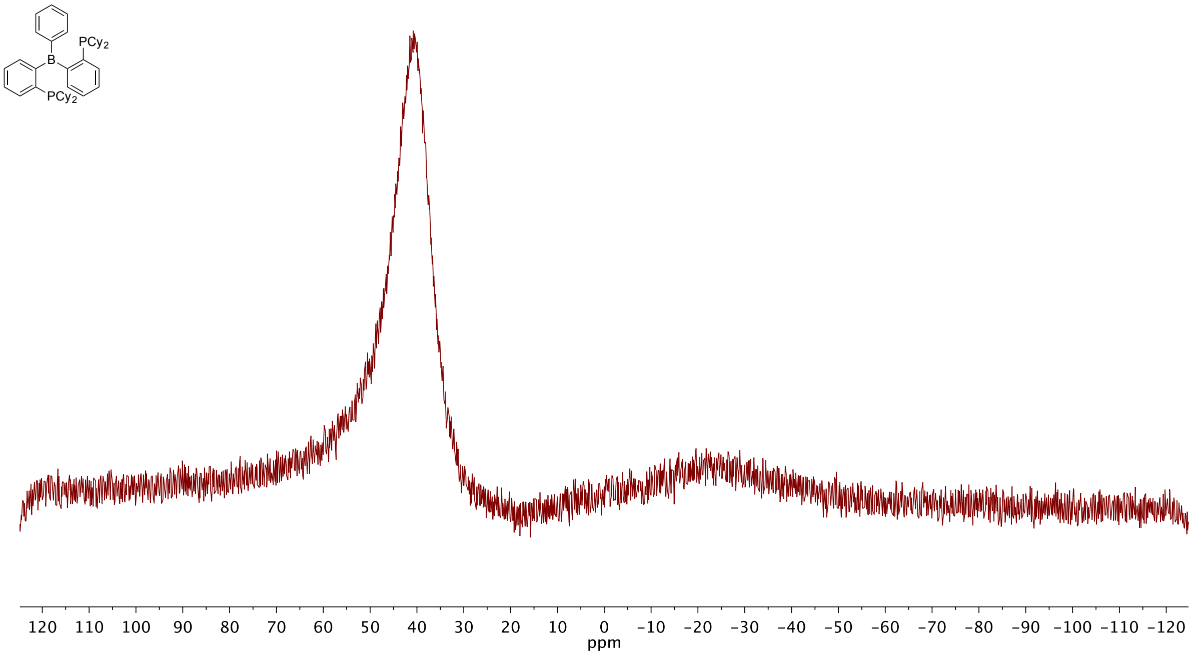

Fig. S4. ^{11}B NMR (128 MHz, dichloromethane-d^{2}) of ^{Cy}**DPB**$^{\mathbf{Ph}}$ after background substraction.

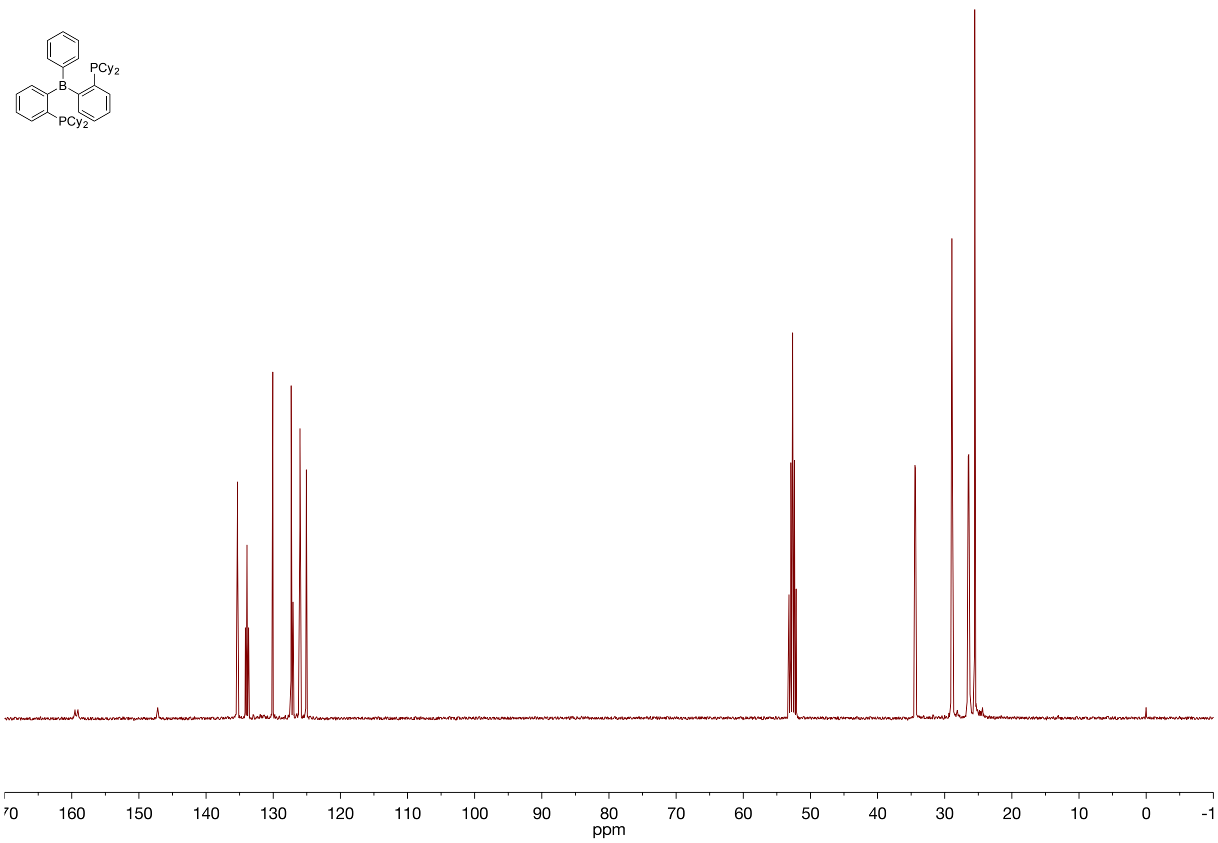

Fig. S5. ^{13}C NMR (101 MHz, dichloromethane-d^{2}) of ^{Cy}**DPB**^{Ph}.

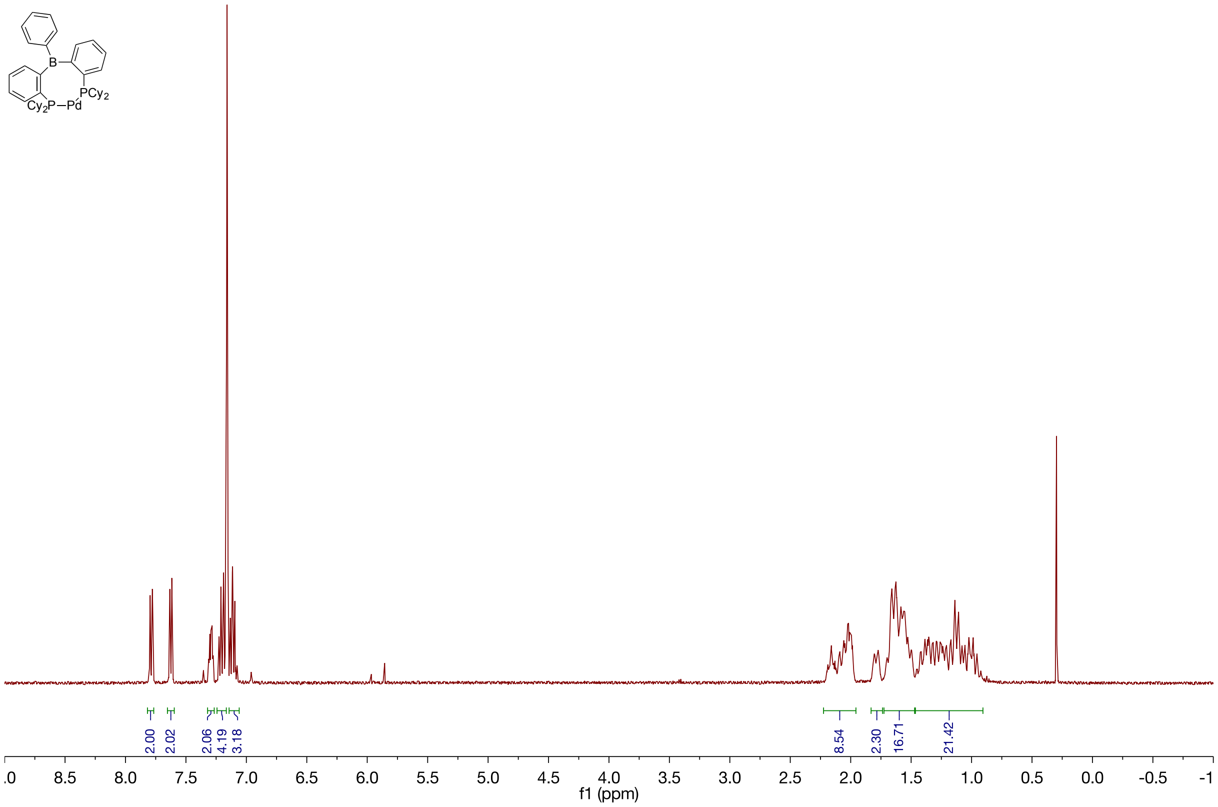

Fig. S6. ^{1}H NMR (400 MHz, benzene-d^{6}) of **8**.

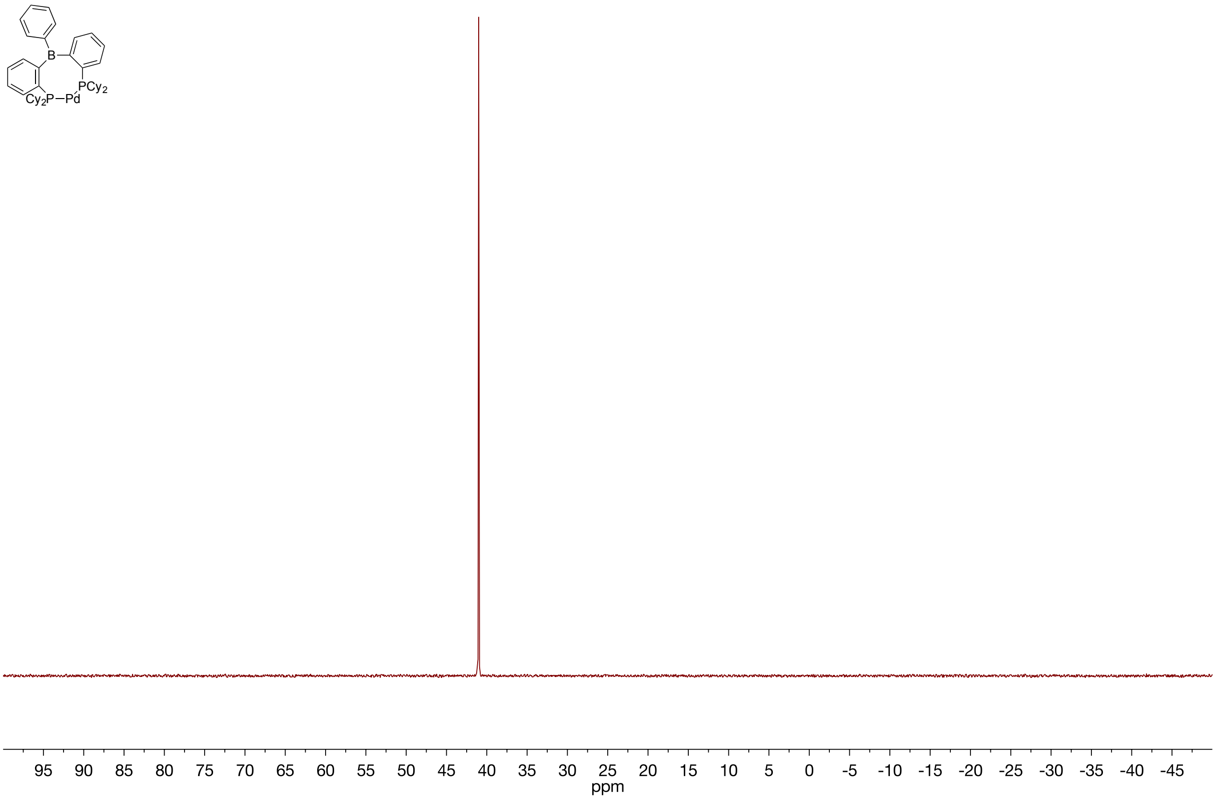

Fig. S7. ^{31}P NMR (162 MHz, benzene-d^{6}) of **8**.

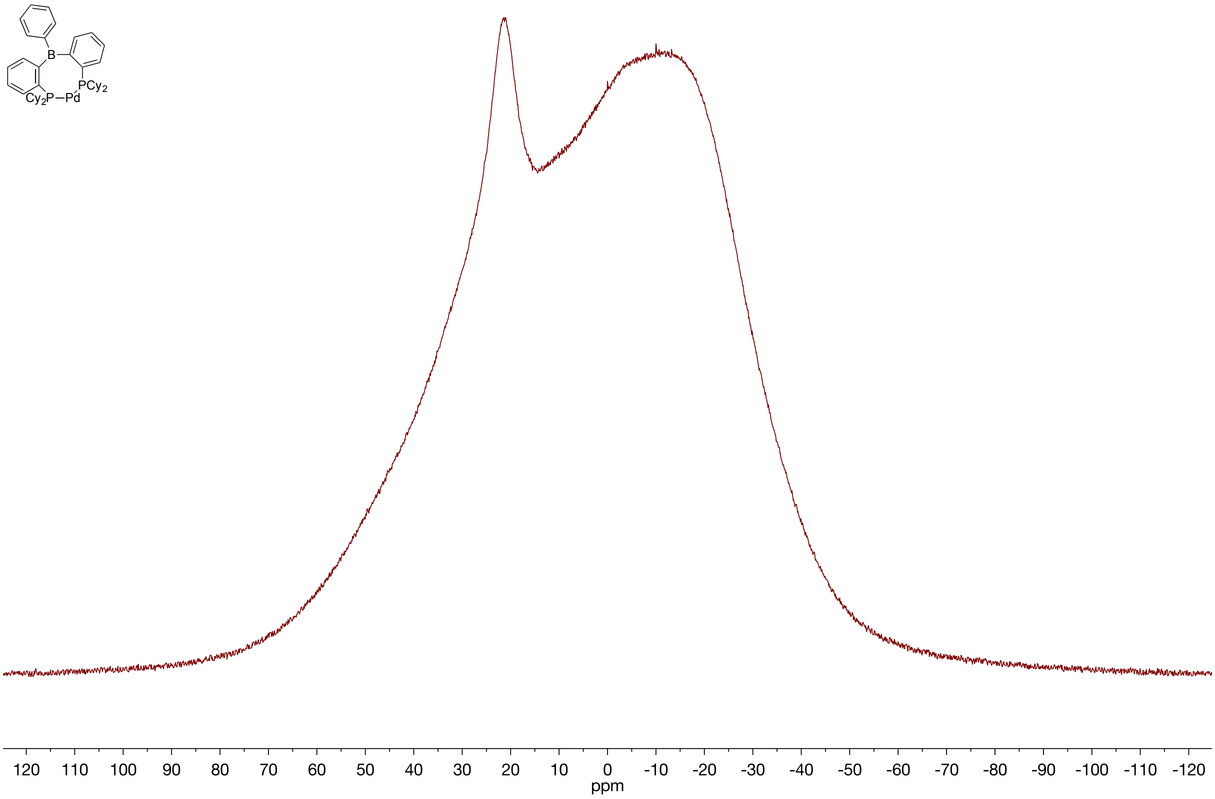

Fig. S8. ^{11}B NMR (128 MHz, benzene-d^{6}) of **8**.

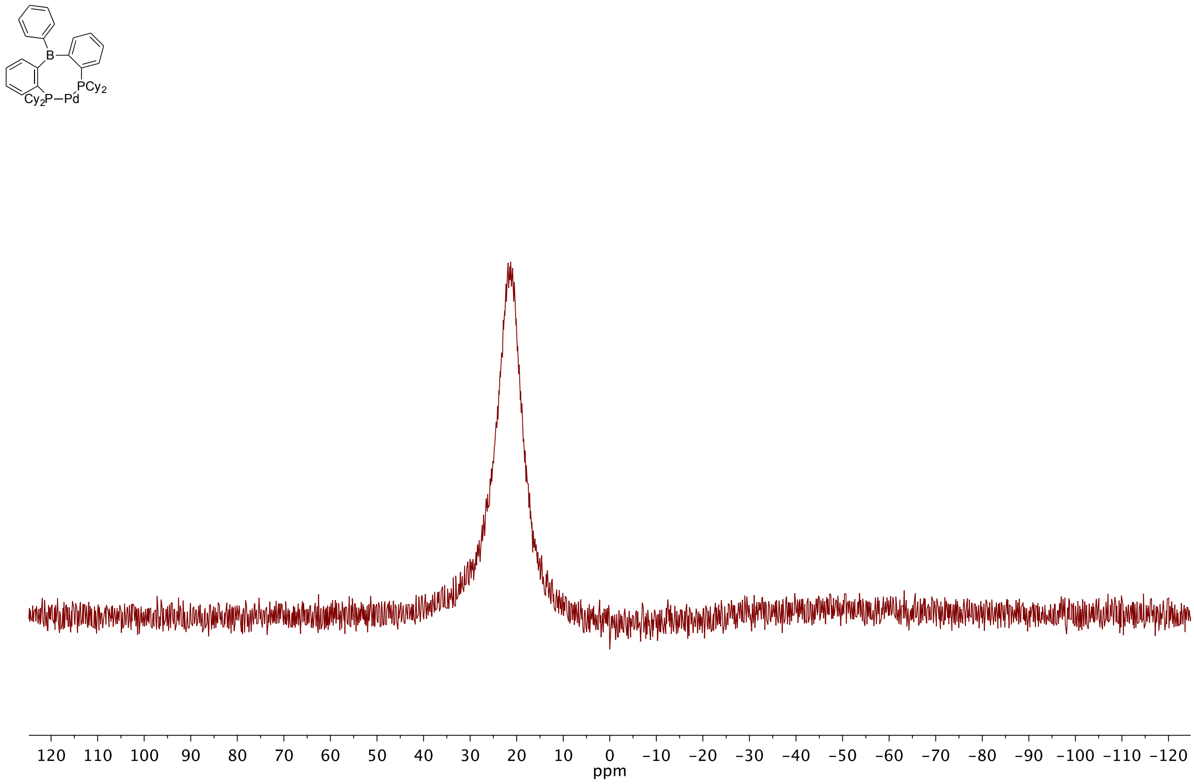

Fig. S9. ^{11}B NMR (128 MHz, benzene-d^{6}) of **8** after background substraction.

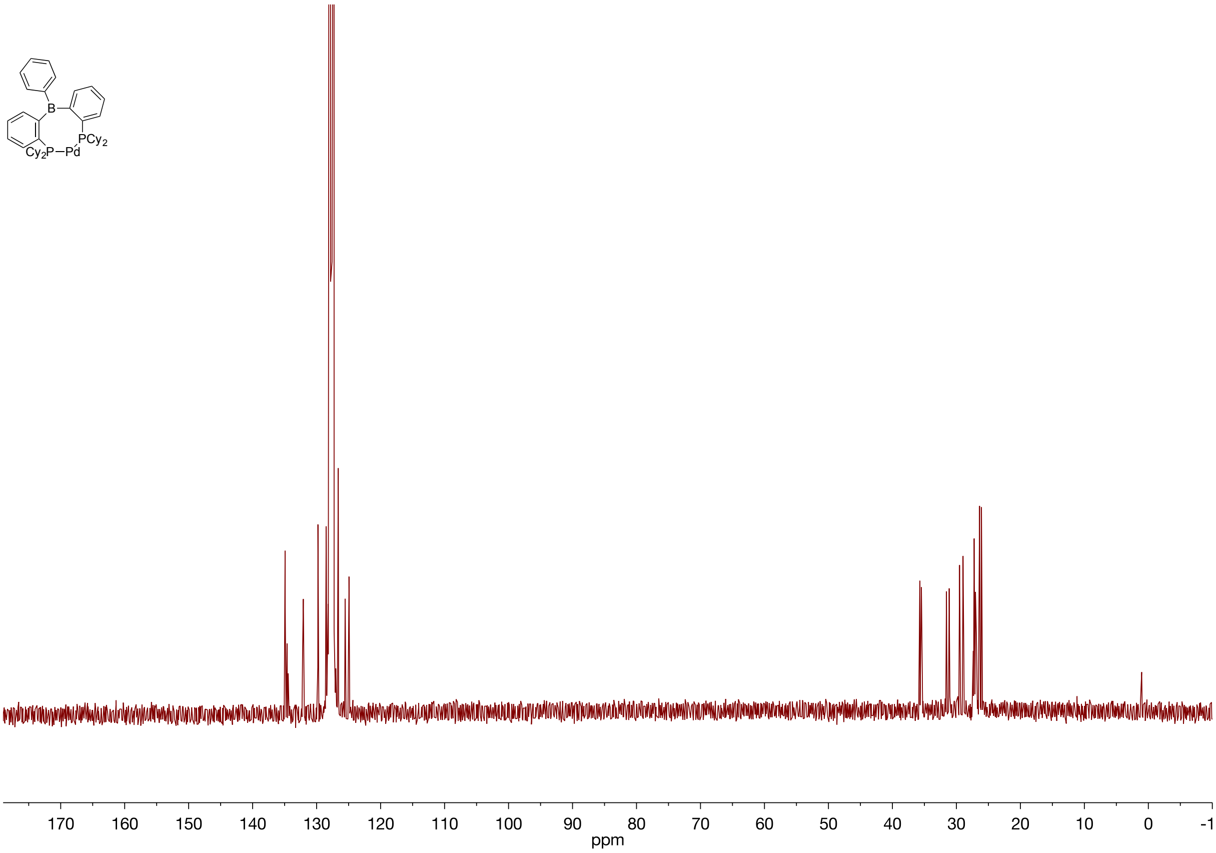

Fig. S10. ^{13}C NMR (101 MHz, benzene-d^{6}) of **8**.

References

1. Tatsuno, O. S.; Yoshida, T.; Otsuka, S. *Inorg. Synth.* **1990**, *26*, *242.*
2. Murata, M.; Buchwald, S. L., *Tetrahedron* **2004**, *60*, 7397-7403.
3. Fulmer, G. R.; Miller, A. J. M.; Sherden, N. H.; Gottlieb, H. E.; Nudelman, A.; Stoltz, B. M.; Bercaw, J. E.; Goldberg, K. I., *Organometallics* **2010**, *29*, 2176.
4. SAINT, Version 6.45. Bruker AXS Inc., Madison, Wisconsin, USA, 2003.
5. SADABS. Bruker AXS Inc., Madison, Wisconsin, USA, 2001.
6. Dolomanov, O. V.; Bourhis, L. J.; Gildea, R. J.; Howard, J. A. K.; Puschmann, H. *J. Appl. Cryst.*, **2009**, *42*, 339.
7. Sheldrick, G. M. *Acta Cryst*., **2008**, *A64*, 112.
8. Sheldrick, G. M. *Acta Cryst.*, **2015**, *C71*, 3.
9. TURBOMOLE V6.5 2013, a development of University of Karlsruhe and Forschungszentrum Karlsruhe GmbH, 1989-2007.
